# Baduanjin Qigong exercise intervention improves subjective and objective cognitive functioning: the moderation and mediation effects of sleep

**DOI:** 10.1002/alz.093211

**Published:** 2025-01-09

**Authors:** Yi Yang, Haoer Shi, Jianghong Liu

**Affiliations:** ^1^ University of Pennsylvania, Philadelphia, PA USA

## Abstract

**Background:**

Baduanjin exercise showed beneficial effects on cognition among older adults, yet the underlying mechanisms are not well understood. We aimed to further examine potential mediation or moderation roles of sleep improvements on the intervention effects in cognition.

**Method:**

In our 10‐week intervention trial with control group, a total of 78 (intervention: n = 47, control: n = 31) older Chinese American adults in a senior daycare center were included. Objective cognition was measured by the computerized memory test. Subjective cognition was assessed by the cognitive function subscale of the Sub‐health Measurement Scale (SHMS). Sleep was measured by Pittsburgh Sleep Quality Index (PSQI). The SPSS PROCESS macro was used for moderation and mediation analyses, with the post‐pre differences in sleep and cognition used as moderator or mediator and outcome, respectively.

**Result:**

Compared to the control group, the Baduanjin Qigong intervention significantly predicted improvements in subjective and objective cognition for poor sleepers, while having no effect on good sleepers. Improvements in sleep quality significantly mediated the intervention effects on subjective cognition among populations with higher education (indirect effect: *β* = ‐.320, CI: [‐.717, ‐.041]) and less exercise (indirect effect: *β* = ‐.133, CI: [‐.573, .259]), while the direct effect of intervention remained significant.

**Conclusion:**

In conclusion, Baduanjin exercise improved cognitive functioning in older Chinese American immigrants, particularly among those with poor sleep. Sleep quality played a mediating role, especially among individuals with higher education and less exercise. Our results shed light on future interventions in helping identify targeted mediating/moderating factors of Baduanjin interventions.

